# Synthesis and Characterization of Celecoxib Derivatives as Possible Anti-Inflammatory, Analgesic, Antioxidant, Anticancer and Anti-HCV Agents †

**DOI:** 10.3390/molecules18033595

**Published:** 2013-03-21

**Authors:** Ş. Güniz Küçükgüzel, İnci Coşkun, Sevil Aydın, Göknur Aktay, Şule Gürsoy, Özge Çevik, Özlem Bingöl Özakpınar, Derya Özsavcı, Azize Şener, Neerja Kaushik-Basu, Amartya Basu, Tanaji T. Talele

**Affiliations:** 1 Department of Pharmaceutical Chemistry, Faculty of Pharmacy, Marmara University, Haydarpaşa, 34668 İstanbul, Turkey; E-Mails: coskun.inci@yahoo.com (İ.C.); sevil.aydin@marmara.edu.tr (S.A.); 2 Department of Pharmacology, Faculty of Pharmacy, Inonu University, 44280 Malatya, Turkey; E-Mails: gaktay@inonu.edu.tr (G.A.); sulegursoy@hotmail.com (Ş.G.); 3 Department of Biochemistry, Faculty of Pharmacy, Marmara University, Haydarpaşa, 34668 İstanbul, Turkey; E-Mails: dagdevirenozge@gmail.com (Ö.Ç.); ozlem.bingol@marmara.edu.tr (Ö.B.Ö.); derya.ozsavci@marmara.edu.tr (D.Ö.); azize.sener@marmara.edu.tr (A.Ş.); 4 Department of Biochemistry, Faculty of Pharmacy, Cumhuriyet University, 58140 Sivas, Turkey; 5 Department of Biochemistry and Molecular Biology, UMDNJ-New Jersey Medical School, 185 South Orange Avenue, Newark, NJ 07103, USA; E-Mails: kaushik@umdnj.edu (N.K.-B.); basu.amartya@gmail.com (A.B.); 6 Department of Pharmaceutical Sciences, College of Pharmacy and Allied Health Professions, St. John’s University, Jamaica, NY 11439, USA; E-Mail: talelet@stjohns.edu

**Keywords:** anti-inflammatory, anticancer, celecoxib, hepatitis C NS5B, sulfonylthiourea

## Abstract

A series of novel *N*-(3-substituted aryl/alkyl-4-oxo-1,3-thiazolidin-2-ylidene)-4-[5-(4-methylphenyl)-3-(trifluoromethyl)-1*H*-pyrazol-1-yl]benzenesulfonamides **2a**–**e** were synthesized by the addition of ethyl α-bromoacetate and anhydrous sodium acetate in dry ethanol to *N*-(substituted aryl/alkylcarbamothioyl)-4-[5-(4-methylphenyl)-3-(trifluoro-methyl)-1*H*-pyrazol-1-yl]benzene sulfonamides **1a**–**e**, which were synthesized by the reaction of alkyl/aryl isothiocyanates with celecoxib. The structures of the isolated products were determined by spectral methods and their anti-inflammatory, analgesic, antioxidant, anticancer and anti-HCV NS5B RNA-dependent RNA polymerase (RdRp) activities evaluated. The compounds were also tested for gastric toxicity and selected compound **1a** was screened for its anticancer activity against 60 human tumor cell lines. These investigations revealed that compound **1a** exhibited anti-inflammatory and analgesic activities and further did not cause tissue damage in liver, kidney, colon and brain compared to untreated controls or celecoxib. Compounds **1c** and **1d** displayed modest inhibition of HCV NS5B RdRp activity. In conclusion, *N*-(ethylcarbamothioyl)-4-[5-(4-methylphenyl)-3-(trifluoromethyl)-1*H*-pyrazol-1-yl]benzenesulfonamide (**1a**) may have the potential to be developed into a therapeutic agent.

## 1. Introduction

Arachidonic acid is an unsaturated fatty acid liberated from phospholipids of cell membranes. Phospholipid hydrolyses cause arachidonic acid release. The eicosanoids are formed by two different routes; namely via the cyclooxygenase (COX) and lipoxygenase (LOX) pathways. Leukotrienes, derived from the lipoxygenase pathway, are responsible for allergy, inflammation and other pathological situations. Cyclic endoperoxides, derived from cyclooxygenase pathway, are converted to derivatives of prostacyclin, prostaglandin E, prostaglandin F_2__α_, tromboxane A_2_ under the action of several enzymes. Prostacyclins and prostaglandins (PG) are major mediators of hyperalgesia which means sensitization of central neurons and an increased response to a painful stimulus. Hyperalgesia is induced by non-steroidal anti-inflammatory drugs (NSAIDs) with the inhibition of cyclooxygenases. Thus, the production of prostaglandin and prostacyclin is hindered [1].

There are two main types of cyclooxygenase that are found in the human body. The enzyme known as COX-3, a COX-1 variant, take place in cerebral cortex and is particularly sensitive to parasetamol. COX-1 is expressed in tissues. In contrast to COX-1, COX-2 is important has a role in ovulation, birth processes and inflammation. The other difference between COX-1 and COX-2 is tissue expression. COX-1 is constitutively expressed in almost all tissues. COX-2 expression is highly restricted, for instance the tissues in patients with malignant and premalignant tumors express increased levels of COX-2.

Factors contributing to chronic inflammation appear to be associated with increased risk of epithelial cancer. The positions where inflammation occurs, cytocine based COX-2 induction results in prostaglandin increases. The role of COX-2 enzyme in aspects of carcinogenesis like proliferation and apoptosis is also effective. Inflammation induced by COX-2 in proliferation mainly depends on the production of prostaglandins. This mechanism of COX-2 in carcinogenesis causes cancer. In addition, COX-2 plays a role in the early stages of hepatocarcinogenesis [2,3,4].

NSAIDs are known as targets of COX in arachidonic acid metabolism and are useful clinical therapeutics for the treatment of inflammation, pain and inflammation-related disorders. Epidemiologic studies have shown that regular use of NSAIDs may reduce the risk of cancer among patients. Furthermore, some NSAIDs have anticancer activities *in vitro*. However, the mechanisms that lead to inhibition of polyp formation are not clear. Researchers have stated that the antitumor activities depend on the inhibition of prostaglandins [5,6]. It is also stated that NSAIDs are responsible for the regulation of normal apoptosis in colorectal polyps and in several cancer cell lines which have lost their polyposis coli gene function [7].

Oxidative stress can be caused by an elevation in the concentration of reactive oxygen species (ROS) [8]. ROS react with any cellular structure and can initiate cell death by necrosis or apoptosis [9,10,11]. Thus, enhanced oxidative stress can caused tissue damage. A large number of drugs and compounds can stimulate the generation of ROS and increase tissue damage [11].

**Figure 1:**
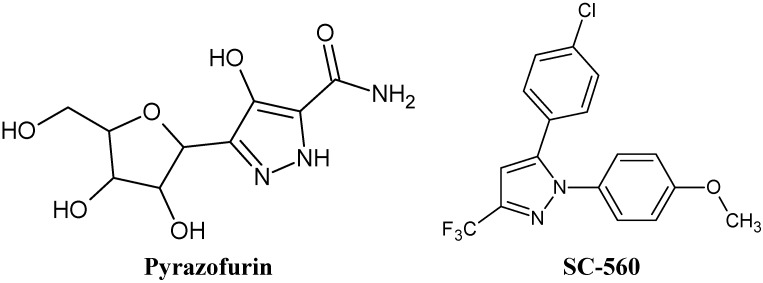
Pyrazofurin and SC-560.

Besides, the presence of a pyrazole moiety and potent activity make pyrazofurin (Figure 1) a useful precursor to synthesize new compounds having antiviral activity. Researchers have reported that the compounds including pyrazole moieties are effective against the HCV virus [12,13,14,15]. The compound SC-560 (Figure 1) as a celecoxib analog, is a selective inhibitor of COX-1 and COX-2. Because of the efficacy against HCV, SC-560 and its derivatives are drug candidates for further studies [16].

Celecoxib, a selective potent COX-2 inhibitor, is currently Food and Drug Administration–approved for chemoprevention in familial adenomatous polyposis based on demonstrable inhibition of colon polyp formation, further solidifying the use of this agent in cancer therapy [17] and proliferation of HCC cell lines [18]. In addition, celecoxib was shown to be a gastrointestinally safe anti-inflammatory analgesic agent.

The sulfonylthioureido group has emerged as the most favourable pharmacophore. Sulfonylthioureido and 4-thiazolidinone groups in combination resulted in anticancer [19] and anti-inflammatory [20] activities. Celecoxib [4-[5-(4-methylphenyl)-3-(trifluoromethyl)-1*H*-pyrazole-1-yl]benzene-sulfonamide], which has a sulfonylamide moiety was used as a starting compound for synthesizing new sulfonylthioureas and after the characterization of their structure these sulfonylthioureas were refluxed with alkyl α-halogenoacetates and anhydrous sodium acetate in absolute ethanol to provide sulfonyliminothiazolidine-4-ones.

The structures of the synthesized compounds were elucidated by chemical and spectroscopic methods, and their biological activity was determined. Furthermore, the ulcerogenic and acute toxicity profiles of the synthesized compounds were determined.

## 2. Results and Discussion

### 2.1. Chemistry

**Scheme 1:**
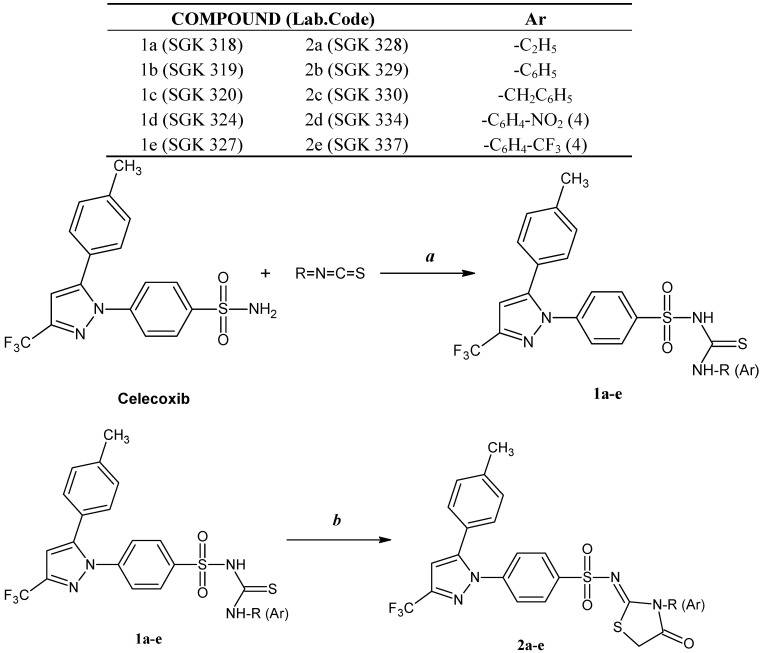
Synthesis of celecoxib derivatives **1a**–**1e**, **2a**–**2e**.

In the present study, we report the synthesis of celecoxib derivatives bearing sulfonylthiourea and 4-thiazolidinone ring systems (Scheme 1). *N*-(substituted carbamothioyl)-4-[5-(4-methylphenyl)-3-(trifluoromethyl)-1*H*-pyrazol-1-yl]benzenesulfonamides **1a**–**e** were prepared with substituted isothiocyanates and anhydrous K_2_CO_3_ in dry acetone starting from celecoxib. *N*-(3-Substituted-4-oxo-1,3-thiazolidine-2-ylidene)-4-[5-(4-methylphenyl)-3-(trifluoromethyl)-1*H*-pyrazol-1-yl]benzenesulfonamide derivatives **2a**–**e** were synthesized by the reaction of ethyl α-bromoacetate and anhydrous sodium acetate in dry ethanol with the sulfonylthioureas **1a**–**e**. Purity of the synthesized compounds was determined by elemental analysis. Structures of these compounds were characterized using FT-IR, ^1^H-NMR, ^13^C-NMR (only **2c**) and HR-MS (**1a** and **2a**) spectral data.

*Reagents*: (**a**) dry acetone, anh. K_2_CO_3_; (**b**) anh. CH_3_COONa, BrCH_2_COOC_2_H_5_, dry ethanol.

The FT-IR spectra of the sulfonylthiourea products **1a**–**e** showed characteristic absorption bands in the thiocarbonyl frequency region at 1122–1134 cm^−1^ and these data is in agreement with the suggested structures. The^1^H-NMR spectra of **1a**–**e** displayed singlets at 9.08–10.75 ppm for the -SO_2_NH group. The ^1^H-NMR spectrum of compound **1b**, taken as a typical example of the prepared series, revealed a singlet corresponding to the –SH proton at 4.20 ppm and –NH peaks were not detected. The thione tautomeric form was converted into the thiol tautomer due to the use of DMSO-d*_6_*. Strong deshielding of this thiol proton can be explained by hydrogen bond formation. The –SO_2_NH proton of **1c **was observed to exchange with deuterium in DMSO-d*_6_*. Also, the signals of the -CH_2_- group appear as two doublets at 4.33 and 4.62 ppm and may result from hindered rotation of the NH-CH_2_- bond in DMSO-d*_6_* solution. A signal at 3.61-4.96 ppm due to -S–CH_2_- confirms the formation of the thiazolidinone ring. The FT-IR spectra of the 4-thiazolidinone derivatives **2a**–**e** were studied and were in agreement with the required structures.

### 2.2. Biological Activity

#### 2.2.1. Anti-inflammatory Activity, Carrageenan-Induced Oedema

At first, the substances were screened for anti-inflammatory activity at 100 mg/kg, p.o. dose. The compounds having more than 15% effectiveness in relieving symptoms, even though some of them are not statistically significant, were considered both for further evaluation of anti-inflammatory activity in two different dose levels (50 and 200 mg/kg) and for screening of their analgesic activities at the dose of 100 mg/kg. Besides, these compounds were also evaluated for their gastric toxicity by determining the lipid peroxidation level and ulcer scores in stomachs. We used celecoxib as a reference compound, because the synthesized compounds were derived from celecoxib. We wanted to investigate the influence of molecular modification on biological activity.

All pharmacological activity results have been summarized in Table 1 and Table 2. As seen in Table 1, the compounds **1a**, **1d**, **1e** and **2d** possessed moderate-to-good anti-inflammatory activity at all doses in all of the measurement intervals. Good inhibition of the second phase of carrageenan-induced oedema was observed for the compounds **1a** and **1d**, suggesting that they interfere with prostaglandin synthesis. Compounds **1e** and **2e** exhibited significant activity, especially at 50 mg/kg doses in the first phase. 

Since the carrageenan oedema has been used in the development of indomethacie, many researchers have adapted this procedure for screening potential anti-inflammatory compounds. Carrageenan-induced oedema is a nonspecific inflammation maintained by the release of histamine, 5-hydroxy-tryptamine, kinins and later by prostaglandins [21]. The inhibitory effect of NSAIDs, such as indomethacin, is usually weak in the first phase (1–2 h), in contrast with their strong inhibition in the second phase (3–4 h) [22]. Furthermore, many studies have demonstrated that the massive production of nitric oxide (NO) and PGE2 via pro-inflammatory proteins inducible nitric oxide synthase (iNOS) and COX-2, respectively, plays an important pathophysiological role in the development of carrageenan-induced thermal hyperalgesia and paw edema [23,24].

There was a noticeable anti-inflammatory activity at 50 mg/kg dose only in compounds **1a**, **1e** and **2d**. Especially, the compound **2d** showed the most remarkable inhibition (48.9%, *p* < 0.01) at 50 mg/kg dose. Moreover, the activity of the compound **2d** at 50 mg/kg dose was more prominent than the ones seen with other doses. It has attracted attention with almost equivalent activity to celecoxib. 

**Table 1:** Dose-dependent anti-inflammatory effects of the compounds against carrageenan-induced hind paw edema model in mice in different doses.

Compounds	Dose mg/kg (per os)	Swelling in thickness (× 10^−2^ mm) ± SEM			
		(percent inhibitory activity)			
		90 min	180 min	270 min	360 min
**Control**					
		72.0 ± 6.6	92.0 ± 8.1	121.0 ± 9.8	129.0 ± 7.8
**1a**	100	58.1 ± 6.6 (19.4)	72.9 ± 11.9 (19.7)	72.7 ± 16.7 (39.5)	77.9 ± 11.7 ** (39.7)
	50	70.0 ± 6.5 (2.7)	72.0 ± 5.1 (21.7)	90.0 ± 9.3 * (25.6)	100.0 ± 6.5 * (22.4)
	200	54.6 ± 7.9 (25.0)	74.2 ± 8.2 (19.3)	117.5 ± 9.8 (2.9)	97.4 ± 5.6 ** (24.5)
**1d**	100	95.9 ± 10.2	100.6 ± 5.8 (18.3)	110.7 ± 11.0 (8.5)	88.7 ± 9.3 * (31.2)
	50	70.0 ± 4.7 (2.7)	77.0 ± 2.0 (16.3)	112.0 ± 7.3 (7.4)	125.0 ± 5.9 (3.1)
	200	45.9 ± 10.9 (36.2)	65.3 ± 6.4 (29.0)	98.2 ± 9.8 (19.0)	91.0 ± 3.3 ** (29.5)
**1e**	100	88.4 ± 2.6	83.9 ± 3.4 (8.8)	88.7 ± 11.4 (26.7)	83.4 ± 9.6 ** (35.4)
	50	47.0 ± 3.3 * (34.7)	57.0 ± 6.2 ** (38.0)	92.0 ± 1.2 * (23.9)	106.0 ± 6.7(17.8)
	200	76.9 ± 5.7	83.0 ± 6.8	110.5 ± 7.5	103.7 ± 5.2 * (19.6)
**2c**	100	83.4 ± 8.7	90.6 ± 11.7	91.7 ± 16.7 (24.2)	118.2 ± 7.1 (8.3)
	50	65.0 ± 6.1 (9.7)	74.0 ± 8.8 (19.5)	95.0 ± 10.0 (21.4)	110.0 ± 7.1 (14.7)
	200	69.5 ± 8.3 (3.4)	75.6 ± 8.3 (17.8)	133.2 ± 14.3	120.1 ± 5.3 (6.9)
**2d**	100	73.2 ± 11.1	100.6 ± 5.8	98.2 ± 16.7 (21.6)	120.9 ± 9.3 (6.3)
	50	47.0 ± 3.3 * (34.7)	47.0 ± 3.7 ** (48.9)	89.0 ± 6.5 * (26.4)	110.0 ± 6.7 (14.7)
	200	80.6 ± 9.4	86.0 ± 9.4 (6.5)	121.0 ± 12..8	108.6 ± 5.6 (15.8)
Celecoxib	25	57.0 ± 6.9 (20.8)	42.0 ± 3.3 ** (54.3)	94.0 ± 10.4 (22.3)	103.0 ± 7.5 * (20.1)

* *p* < 0.05; ** *p* < 0.01; *** *p* < 0.001; significant from control (n = 4–5).

#### 2.2.2. Analgesic Activity

The analgesic activities of compounds **1a**, **1d**, **1e**, **2c** and **2d **were tested according the Koster test compared to celecoxib and aspirin (ASA) (Table 2). In this study, the synthesized compounds were obtained from celecoxib and there were no guarantee that our compounds would have no ulcerogenic potential. Therefore, we used ASA as a positive control for ulcerogenic activity whereas celecoxib were also used as negative control.

The analgesic activity of the compounds was studied using the acetic acid-induced writhing test in mice and expressed as “mean increase in latency after drug administration ± SEM” relative to control and percentage inhibition in writhing reflex. Although the compounds **2c **and **2d **showed some activity, it is not statistically significant. Among the compounds, *N*-(ethylcarbamothioyl)-4-[5-(4-methylphenyl)-3-(trifluoromethyl)-1*H*-pyrazol-1-yl] benzenesulfonamide (**1a**) attracted attention with higher analgesic activity than celecoxib with a percentage inhibition valus of 67.8 at 100 mg/kg dose level. These observations allow us to conclude that compounds having aliphatic sulfonylthiourea groups possessed higher activity than compounds having aromatic sulfonylthiourea groups and sulfonyliminothiazolidine-4-ones.

**Table 2:** Analgesic effects of the test compounds at a 100 mg/kg dose, against acetic acid-induced abdominal constriction test, ulcer score and the lipid peroxidation levels in stomach of mice.

Compounds	Writhing test (Mean ± SEM) (% inh.)	Ulcer score	Lipid peroxidation
		(200 mg/kg)	(nmol TBARS/g wet weight)
Control	17.4 ± 3.7	0/5	387.7 ± 27.9
Celecoxib (25 mg/kg)	8.0 ± 1.3 (54) *	0/5	417.8 ± 23.3
ASA (200 mg/kg)	4.4 ± 0.9 (74.7) **	3/5	436.1 ± 17.1
**1a**	5.6 ± 0.9 (67.8) **	0/5	387.7 ± 12.8
**1d**	14.0 ± 3.0 (19.5)	0/5	429.2 ± 23.2
**1e**	24.0 ± 3.0 (−37.9)	0/5	406.9 ± 27.0
**2c**	11.8 ± 1.4 (32.2)	0/5	330.4 ± 9.7
**2d**	12.0 ± 2.6 (31)	0/5	350.2 ± 9.9

* *p* < 0.05; ** *p* < 0.01; significant from control.

#### 2.2.3. Acute Ulcerogenesis

The compounds, which were screened for analgesic activity, were further screened for their acute ulcerogenic risk. All tested compounds were found to be safer as gastric lesion risks at high dose (200 mg/kg. p.o) when compared to aspirin (Table 2). Morever, gastric safety of celecoxib has been identified ast not changed by molecular modification in this study.

#### 2.2.4. Acute Toxicity

None of the test compounds used in the pharmacologic study produced lethal effects and did not induce any significant behavioral modification at the employed doses during the observation period.

#### 2.2.5. Antioxidant Activity, Lipid Peroxidation in Stomach

It is well established that reactive oxygen species have a decisive role in inflammatory conditions [8]. It has also been reported that oxidative stress is important for gastrointestinal ulceration [8,25]. The compounds having antioxidant activity besides anti-inflammatory/analgesic activity may offer a viable route to safer anti-inflammatory/analgesic agents. The obtained lipid peroxidation (LPO) values revealed that the ulcerogenic effect of the compounds were appreciably less than ASA.

#### 2.2.6. Tissue Damage/Antioxidant Effects of Compound **1a** in Various Tissues

The antioxidant/ toxic effects of compound **1a**, the best analgesic activity observed were examined by using some of oxidative stress parameters on other tissues except for the stomach. The aim of this study was to evaluate the effect of the compound **1a**, which was orally administered to mice at doses of 100 mg/kg and 200 mg/kg, in two different doses on LPO, glutathione (GSH) levels, myeloperoxidase (MPO) and superoxide dismutase (SOD) activities of various tissues (liver, kidney, colon and brain) (Table 3, Table 4, Table 5 and Table 6) [26,27,28]. According to our result, we didn’t find any differences in oxidative stress parameters between groups (*p* > 0.05). This study showed that compound **1a** has no effects in tissue (liver, kidney, colon and brain) damage. Thus, this compound may safely use as therapeutic agent in the future.

**Table 3:** Oxidative stress parameters in liver.

Parameters	Control	Celecoxib	Comp. 1a	Control	Celecoxib	Comp. 1a
			(100 mg/kg)			(200 mg/kg )
LPO (nmol/gtissue)	8.80 ± 0.82	9.56 ± 1.80	9.78 ± 1.30	15.28 ± 2.06	11.74 ± 2.25	13.72 ± 3.05
MPO (U/gtissue)	0.54 ± 0.31	0.63 ± 0.30	0.50 ± 0.28	0.21 ± 0.01	0.19 ± 0.06	0.15 ± 0.08
GSH (µmol/g tissue)	24.06 ± 4.07	20.58 ± 4.25	22.68 ± 1.20	21.80 ± 6.40	22.14 ± 1.04	24.58 ± 4.47
SOD (U/g tissue)	18.67 ± 14.70	22.78 ± 14.45	21.15 ± 9.98	10.36 ± 5.22	13.60 ± 11.98	11.72 ± 5.10

**Table 4:** Oxidative stress parameters in kidney.

Parameters	Control	Celecoxib	Comp. 1a	Control	Celecoxib	Comp. 1a
			(100 mg/kg)			(200 mg/kg )
LPO (nmol/g tissue)	13.42 ± 3.43	11.12 ± 2.41	10.96 ± 2.01	15.18 ± 2.15	11.30 ± 2.52	12.56 ± 2.96
MPO (U/g tissue)	4.97 ± 1.87	3.36 ± 1.57	3.18 ± 2.66	3.59 ± 1.37	4.75 ± 1.59	4.59 ± 1.01
GSH (µmol/g tissue)	20.76 ± 5.60	22.24 ± 4.17	17.02 ± 5.23	18.77 ± 5.52	21.68 ± 5.56	18.26 ± 3.28
SOD (U/g tissue)	15.09 ± 5.94	17.79 ± 7.47	20.26 ± 8.71	14.14 ± 8.81	16.83 ± 9.71	15.37 ± 7.33

**Table 5:** Oxidative stress parameters in colon.

Parameters	Control	Celecoxib	Comp. 1a	Control	Celecoxib	Comp. 1a
			(100 mg/kg)			(200 mg/kg )
LPO (nmol/g tissue)	7.25 ± 0.27	7.12 ± 0.26	8.66 ± 2.23	8.71 ± 1.86	10.48 ± 2.69	11.04 ± 2.11
MPO (U/gtissue)	0.28 ± 0.19	0.52 ± 0.26	0.27 ± 0.13	0.32 ± 0.10	0.46 ± 0.26	0.38 ± 0.14
GSH (µmol/g tissue)	22.98 ± 2.95	28.06 ± 1.45	27.22 ± 2.41	26.42 ± 7.48	26.42 ± 3.46	25.16 ± 2.76
SOD (U/g tissue)	20.72 ± 7.28	16.85 ± 5.24	16.56 ± 4.13	22.41 ± 6.42	19.04 ± 5.60	21.17 ± 4.15

**Table 6:** Oxidative stress parameters in brain.

Parameters	Control	Celecoxib	Comp. 1a	Control	Celecoxib	Comp. 1a
			(100 mg/kg)			(200 mg/kg )
LPO (nmol/g tissue)	7.53 ± 0.58	8.18 ± 2.16	8.36 ± 2.61	8.42 ± 1.74	8.26 ± 1.01	9.40 ± 2.08
MPO (U/gtissue)	0.33 ± 0.21	0.60 ± 0.23	0.73 ± 0.32	1.42 ± 0.37	1.48 ± 0.46	1.95 ± 0.60
GSH (µmol/g tissue)	18.88 ± 4.13	22.36 ± 2.45	18.7 ± 5.52	16.26 ± 2.81	20.2 ± 3.26	18.74 ± 3.03
SOD (U/g tissue)	20.84 ± 4.83	24.84 ± 10.65	19.17 ± 7.41	20.19 ± 4.86	20.41 ± 13.73	19.22 ± 10.76

#### 2.2.7. Anticancer Activity

The selected compound *N*-(ethylcarbamothioyl)-4-[5-(4-methylphenyl)-3-(trifluoromethyl)-1*H*-pyrazol-1-yl]benzenesulfonamide (**1a**) was screened by the National Institutes of Health (NIH) for anticancer activity against 60 human tumor cell lines. Most of the compounds in the series of structures submitted include one or more functional groups that have been found troublesome to the development of successful drug candidates. In addition, the selection criteria guidance available at the following DTP web site: http://www.dtp.nci.nih.gov/docs/misc/common_files/guidelines.html [29]. For NCI criteria, compounds, which reduce the growth of any one of the cell lines to approximately 32% or less, are passed on for evaluation in the full panel of cell lines over a 5-log dose range. Compound **1a** exhibited significant cytotoxicity in melonoma Malm-3M cells (150.58%). 

#### 2.2.8. Effect on HCV NS5B Polymerase Enzyme Inhibition

The ability of the compounds to inhibit HCV NS5B RdRp activity was investigated *in vitro* employing poly rA-U_12_ template-primer as described in the Experimental section [30,31,32]. The compounds **1a**–**e** and **2a**–**e** were reconstituted in DMSO as 50 mM stocks, and serially diluted in DMSO to obtain working stocks. Wedelolactone, a known inhibitor of HCV NS5B activity [32], was employed as a reference compound. Preliminary screening was carried out at 100 μM to identify a wider range of compounds. Celecoxib, the parent molecule, included in this investigation for comparison with its derivatives, exhibited the lowest activity against NS5B of ~10%. All other compounds except **2a** and **2e** exhibited varying degrees of inhibition ranging from ~19% to 83% at 100 μM concentration (Table 7). Of these, compounds **1c** and **1d **exhibited modest inhibition, with IC_50_ values of ~36 μM and 46 μM.

**Table 7:** Anti-HCV NS5B RdRp activity of celecoxib derivatives *in vitro* *.

Compound	Ar/R	Anti-NS5B Activity	IC_50_ (µM)
		(% Inh., 100 µM)	
1a	-C_2_H_5_	49.4	N.D.
1b	-C_6_H_5_	53.5	N.D.
1c	-CH_2_C_6_H_5_	82.3	36.2 ± 1.2
1d	-C_6_H_4_-(4-NO_2_)	68	45.5 ± 1.2
1e	-C_6_H_4_-(4-CF_3_)	14.6	N.D.
2a	-C_2_H_5_	8.2	N.D.
2b	-C_6_H_5_	N.D.	N.D.
2c	-CH_2_C_6_H_5_	19.4	N.D.
2d	-C_6_H_4_-(4-NO_2_)	31.3	N.D.
2e	-C_6_H_4_-(4-CF_3_)	9.4	N.D.
Celecoxib		9.5	

* Percent inhibition was determined at 100 µM concentration of the indicated compound and represents an average of at least two independent measurements in duplicate. The IC_50_ values of the compounds were determined from dose-response curves employing 8–12 concentrations of each compound in duplicate in two independent experiments. Curves were fitted to data points using nonlinear regression analysis and IC_50_ values were interpolated from the resulting curves using GraphPad Prism 3.03 software. (N.D. = not determined.).

To investigate the potential binding mode of celecoxib derivatives to HCV NS5B, we performed molecular modeling studies with the most potent compounds **1c** and **1d** using the Glide docking software, as previously described [32]. To evaluate which of the five allosteric pockets these inhibitors tightly bind to, docking experiments were conducted against each of the five reported NS5B allosteric pockets such as TP-I (PDB ID: 2XWY) [33], TP-II (PDB ID: 3FRZ) [34], palm pocket (PP)-I (PDB ID: 3TYV) [35], PP-II (PDB ID: 3FQL) [36] and PP-III, that significantly overlaps with pocket PP-II (large grid box created around HCV-796 coordinates to obtain docking pose at pocket PP-III). The binding energy (XP-Glide score) of compound **1c** and **1d** was found to be −6.98 and −6.57, −7.70 and −6.50, −9.45 and −9.44, −5.60 and −5.75, −5.74 and −5.45, at TP-I, TP-II, PP-I, PP-II and PP-III, respectively. The relatively more negative Gscore in PP-I versus other pockets indicated a better fit of compounds **1c** and **1d** in PP-I, thus suggesting that PP-I may be the potential binding site for celecoxib derivatives.

**Figure 2:**
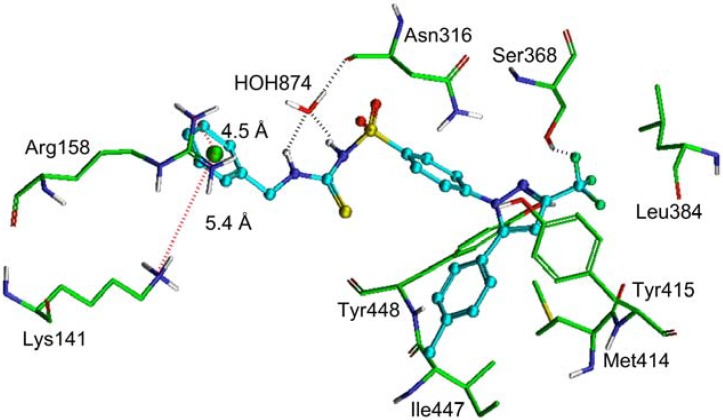
XP-Glide predicted binding mode of compound **1c **within palm pocket-I of NS5B. Important amino acids contacting compound **1c** are depicted as stick model with the atoms colored as carbon—green, hydrogen—white, nitrogen—blue, oxygen—red and sulfur—yellow. Compound **1c** is shown as ball and stick model with the same color scheme as above except carbon atoms are represented in cyan and fluoro atoms in green color. The dotted black line indicates hydrogen bonding interaction whereas the dotted red line indicates cation-pi interaction with distances in Å.

To gain insight into the mechanism of inhibition by celecoxib derivatives, we analyzed the interactions of the docked conformation of potent compound **1c** within PP-I of NS5B (Figure 2). The phenyl ring of the benzyl substituent appeared to establish cation-pi interactions with the positively charged ε-NH_2_ and guanidinium group of Lys141 and Arg158, respectively. Both the -NH groups of thiourea moiety form water-mediated hydrogen bonding interactions with the backbone carbonyl oxygen atom of Asn316 (-NH---OH_2_-874, 1.7 Å; -NH---OH_2_-874, 2.5 Å; H_2_O---O=C-Asn316, 1.9 Å). The phenyl ring attached to the sulfonamide group is stabilized by hydrophobic interactions with the side chain of Tyr448. The tolylpyrazole moiety is extensively stabilized by hydrophobic interactions with the side chains of Met414, Tyr415, Ile447 and Tyr448. The trifluoromethyl group forms hydrogen bond with the side chain hydroxyl group of Ser368 (-F---HO-Ser368, 1.8 Å) in addition to hydrophobic interaction with Leu384.

## 3. Experimental

### 3.1. General

All chemical compounds were purchased from Sigma-Aldrich and Fluka. Melting points were taken on Schmelzpunktbestimmer SMP II apparatus and are uncorrected. IR spectra were recorded on a Shimadzu FTIR-8400S instrument. ^1^H-NMR and ^13^C-NMR spectra were obtained on a Bruker AVANCE 500 and Varian Mercury 400 instrument and chemical shifts were reported in ppm (δ). High resolution electron impact mass spectra were recorded on a Jeol JMS-700 instrument. The microwave assisted reactions were carried out in a household microwave oven (MW 570 manufactured by Kenwood Corporation, maximum power output of 900 W). Merck silica gel 60 F254 plates were used for analytical TLC. The purities of the synthesized compounds were checked using thin layer chromatography in petroleum ether (PE): acetone (λ = UV 254 nm, t = 25 °C).

### 3.2. Chemistry

#### 3.2.1. General Procedure for the Synthesis of *N*-(Substituted aryl/alkyl carbamothioyl)-4-[5-(4-methylphenyl)-3-(trifluoromethyl)-1*H*-pyrazol-1-yl]benzenesulfonamides **1a**–**e**

A solution of the appropriate isothiocyanate (0.00275 mol) in dry acetone (5 mL) was added to a stirred mixture of celecoxib (0.0025 mol) and anhydrous potassium carbonate (0.005 mol) in dry acetone (20 mL), and the reaction mixture was heated under reflux for 20–25 h. The progress of reaction was monitored by thin layer chromatography. After completion of the reaction, the reaction mixture dissolved in water and acidified with hydrochloric acid (2 N). The precipitate obtained was filtered off, washed with water, dried and recrystallised twice from ethanol. 

*N-(Ethylcarbamothioyl)-4-[5-(4-methylphenyl)-3-(trifluoromethyl)-1H-pyrazol-1-yl] benzenesulfonamide* (**1a**). White powder, yield 86%, mp: 215–218 °C.^1^H-NMR (400 MHz, DMSO-d*_6_*/TMS) δ ppm: 1.01 (3H, t, *J* = 7.1 Hz, -CH_2_-C**H**_3_); 2.31 (3H, s, Ar-C**H**_3_); 3.67 (2H, m, -C**H**_2_-CH_3_); 7.16–8.51 (10H, m, Ar-**H** ve CSN**H**-CH_2_-CH_3_); 10.75 (1H, s, -SO_2_-N**H**). FT-IR ν _max_. (cm^−1^): 3337 (sulfonylthiourea, -NH); 1345 and 1153 (SO_2_); 1122 (C=S). Anal. for C_20_H_19_F_3_N_4_O_2_S_2_: Found: C, 50.52; H, 4.04; N, 11.77%. Calc: C, 51.27; H, 4.09; N, 11.96% . HRMS–EI: *m/z* calcd for C_20_H_19_F_3_N_4_O_2_S_2_ [M+H]^+^: 469.0974; found: 469.0974. R*_f_*: 0.73 (PE/Acetone, 5:5);

*N-(Phenylcarbamothioyl)-4-[5-(4-methylphenyl)-3-(trifluoromethyl)-1H-pyrazol-1-yl] benzene-sulfonamide* (**1b**). White powder, yield 70%, mp: 251–252 °C. ^1^H-NMR (500 MHz, DMSO-d*_6_*/TMS) δ ppm: 2.29 (3H, s, Ar-C**H**_3_); 4.20 (1H, s, S**H** in thiol form); 6.83–7.83 (14H, m, Ar-**H**); 9.08 (1H, s, -SO_2_-N**H**). FT-IR ν _max_. (cm^−1^): 3655 (-OH, H_2_O); 3340 (sulfonylthiourea, -NH); 1319 and 1172 (SO_2_); 1128 (C=S). Anal. for C_24_H_19_F_3_N_4_O_2_S_2_. H_2_O: Found: C, 54.50; H, 3.52; N, 9.58%. Calc.: C, 53.92; H, 3.96; N, 10.48%. R*_f_*: 0.55 (PE/Acetone, 6:4). 

*N-(Benzylcarbamothioyl)-4-[5-(4-methylphenyl)-3-(trifluoromethyl)-1H-pyrazol-1-yl] benzene-sulfonamide* (**1c**). White powder, yield 73%. mp: 237–240 °C. ^1^H-NMR (400 MHz DMSO-d*_6_*/TMS) δ ppm: 2.30 (3H, s, Ar-C**H**_3_); 4.33 (1H, d, -NH-C**H**_2_, *J* = 6.19 Hz); 4.62 (1H, d, -NH-C**H**_2_, *J* = 6.00 Hz); 7.16–7.78 (15H, m, Ar-**H** ve CSN**H**). FT-IR ν _max_. (cm^−1^): 3612 (-OH, H_2_O); 3360 (sulfonylthiourea, -NH.); 1377 and 1220 (SO_2_); 1124 (C=S). Anal. for C_25_H_21_F_3_N_4_O_2_S_2_. 3 H_2_O: Found: C, 51.72; H, 3.85; N, 9.54%. Calc.: C, 51.36; H, 4.65; N, 9.58%. R*_f_*: 0.74 (PE/Acetone, 5:5). 

*N-[(4-Nitrophenyl)carbamothioyl])-4-[5-(4-methylphenyl)-3-(trifluoromethyl)-1H-pyrazol-1-yl]-benzenesulfonamide* (**1d**). Light yellow powder, yield 62%. mp: 245–248 °C. ^1^H-NMR (500 MHz, DMSO-d*_6_*/TMS) δ ppm: 2.29 (3H, s, Ar-C**H**_3_ ); 7.17–8.07 (14H, m, Ar-**H** ve CSN**H**), 9.82 (1H, s, -SO_2_-N**H**). FT-IR ν _max_. (cm^−1^): 3616 (-OH, H_2_O); 3230 (sulfonylthiourea, -NH.); 1323 and 1166 (SO_2_); 1134 (C=S). Anal. Calc. for C_24_H_18_F_3_N_5_O_4_S_2_ .3 H_2_O: Found: C, 47.12; H, 3.11; N, 11.38%. Calc.: C, 46.83; H, 3.93; N, 11.38%. R*_f_*: 0.48 (PE/Acetone, 5:5). 

*N-[(4-Trifluoromethylphenyl)carbamothioyl])-4-[5-(4-methylphenyl)-3-(trifluoromethyl)-1H-pyrazol-1-yl]benzenesulfonamide* (**1e**). White powder, yield 77%, mp: 228–231 °C. ^1^H-NMR (400 MHz, DMSO-d*_6_*/TMS) δ ppm: 2.29 (3H, s, Ar-C**H**_3_); 7.17–7.94 (14H, m, Ar-**H** ve CSN**H**-Ar); 9.50 (1H, s, -SO_2_-N**H**). FT-IR ν _max_. (cm^−1^): 3676 (-OH, H_2_O); 3343 (sulfonylthiourea, -NH.); 1323 and 1232 (SO_2_); 1128 (C=S). Anal. for C_25_H_18_F_6_N_4_O_2_S_2_. 2 H_2_O: Found C, 48.47; H, 2.91; N, 8.91%. Calc.: C, 48.38; H, 3.57; N, 9.03%. R*_f_*: 0.76 (PE/Acetone, 5:5).

#### 3.2.2. Synthesis of *N*-(3-Substituted aryl/alkyl-4-oxo-1,3-thiazolidin-2-ylidene)-4-[5-(4-methylphenyl)-3-(trifluoromethyl)-1H-pyrazol-1-yl]benzenesulfonamides **2a**–**e**

##### *Method I* (*Thermal*)

To a solution of the appropriate sulfonylthiourea derivative **1a**–**e** (0.001 mol) in absolute ethanol (20 mL) were added ethyl α-bromoacetate (0.0011 mol) and anhydrous sodium acetate (0.002 mol) and the reaction mixture was heated under reflux for 2–4 h. The precipitated solid was filtered, washed with water, dried and recrystallized twice from ethanol.

##### *Method II* (*Microwave Assisted Synthesis for* **2d** *and* **2e**)

A mixture of the appropriate sulfonylthiourea derivative **1a**–**e** (0.001 mol) in absolute ethanol (20 mL), ethyl α-bromoacetate (0.0011 mol) and anhydrous sodium acetate (0.002 mol) was placed in the microwaveoven and irradiated at 270 W for 10–15 min. Then, the reaction mixture was left to cool to room temperature. The precipitated solid was filtered, washed with water, dried and recrystallized twice from ethanol.

*N-(3-Ethyl-4-oxo-1,3-thiazolidin-2-yldene)-4-[5-(4-methylphenyl)-3-(trifluoromethyl)-1H-pyrazol-1-yl]benzenesulfonamide* (**2a**). Brown powder, yield 65%, mp: 282 °C. ^1^H-NMR (400 MHz, DMSO-d*_6_*/TMS) δ ppm: 0.98–1.10 (3H, m, N-CH_2_-C**H**_3_); 2.31 (3H, s, Ar-C**H**_3_); 3.35 (2H, q, N-C**H**_2_-CH_3_); 3.61 (1H, d, S-C**H**_2_, *J* = 7.2 Hz); 3.79 (1H, d, S-C**H**_2_ , *J* = 7.2 Hz); 7.16–8.02 (9H, m, Ar-**H**).; FT-IR ν _max_. (cm^−1^): 1708 (C=O); 1334 and 1234 (SO_2_). Anal. for C_22_H_19_F_3_N_4_O_3_S_2_: Found: C, 51.75; H, 3.53; N, 10.78%. Calc.: C, 51.96; H, 3.77; N, 11.02%. HRMS–EI: *m/z* calcd for C_22_H_19_F_3_N_4_O_3_S_2_ [M]^+.^: 508.0845; found: 508.0860. R*_f_*: 0.47 (PE/Acetone, 6:4).

*N-(3-Phenyl-4-oxo-1,3-thiazolidin-2-yldene)-4-[5-(4-methylphenyl)-3-(trifluoromethyl)-1H-pyrazol-1-yl]benzenesulfonamide* (**2b**). Light red powder, yield 57%, mp: 248–249 °C. ^1^H-NMR (500 MHz, CDCl_3_/TMS) δ ppm: 2.38 (3H, s, Ar-C**H**_3_); 4.05 (2H, s, S-C**H**_2_); 6.74–7.83 (14H, m, Ar-**H**). FT-IR ν _max_. (cm^−1^): 3471 (-OH, H_2_O); 1749 (C=O); 1373 and 1236 (SO_2_). Anal. for C_26_H_19_F_3_N_4_O_3_S_2_. 3 H_2_O: Found: C, 50.63; H, 3.24; N, 9.49%. Calc.: C, 51.14; H, 4.13; N, 9.18%. R*_f_*: 0.58 (PE/Acetone, 6:4).

*N-(3-Benzyl-4-oxo-1,3-thiazolidin-2-yldene)-4-[5-(4-methylphenyl)-3-(trifluoromethyl)-1H-pyrazol-1-yl]benzenesulfonamide* (**2c**). Brown powdes, yield 48%, mp: 250 °C. ^1^H-NMR (400 MHz, DMSO-d*_6_*/TMS) δ ppm: 2.32 (3H, s, Ar-C**H**_3_); 3.35 (2H, s, N-C**H**_2_-Ar); 4.96 (2H, s, S-C**H**_2_); 7.22–7.91 (14H, m, Ar-**H**). ^13^C-NMR (100 MHz, DMSO-d*_6_*/TMS) δ ppm: 21.49 (**C**H_3_ -Ar); 39.55–40.80 (thiazolidinone C-5); 47.51 (**C**H_2_ -Ar); 107.50 (pyrazole C-4); 125.53 (benzenesulfonyl C-3, C-5); 125.91 (benzenesulfonyl C-2, C-6); 127.00 (**C**F_3_); 128.41 (4-methylphenyl C-2, C-6); 128.66 (benzyl C-2, C-6); 129.17 (benzyl C-3, C-5); 129.48 (4-methylphenyl C-3, C-5); 130.15 (benzyl C-4); 134.94 (**C**-SO_2_); 135.10 (4-methylphenyl C-1); 139.81 (benzyl C-1); 139.89 (4-methylphenyl C-4); 143.25 (pyrazole C-5); 143.50 (benzenesulfonyl C-4); 146.07 (pyrazole C-3); 165.34 (**C**=N); 166.35 (**C**=O).FT-IR ν _max_. (cm^−1^): 3362 (-OH, H_2_O); 1707 (C=O); 1336 and 1153 (SO_2_). Anal. for C_27_H_21_F_3_N_4_O_3_S_2_. H_2_O: Found: C, 55.09; H, 3.94; N, 9.52. Calc.: C, 54.56; H, 3.36; N, 9.38%. R*_f_*: 0.49 (PE/Acetone, 7:3).

*N-[3-(4-Nitrophenyl)-4-oxo-1,3-thiazolidin-2-yldene)-4-[5-(4-methylphenyl)-3-(trifluoromethyl)-1H-pyrazol-1-yl]benzenesulfonamide* (**2d**). Light yellow powder, yield 60%, mp: 270–272 °C. ^1^H-NMR (400 MHz, DMSO-d_6_/TMS) δ ppm: 2.38 (3H, s, Ar-C**H**_3_); 4.10 (2H, s, S-C**H**_2_); 6.74–8.32 (13H, m, Ar-**H**). FT-IR ν _max_. (cm^−1^): 1749 (C=O); 1348 and 1147 (SO_2_). Anal. for C_26_H_18_F_3_N_5_O_5_S_2_: Found: C, 51.08; H, 3.14; N, 11.90%. Calc.: C, 51.91; H, 3.02; N, 11.64%. R*_f_*: 0.67 (PE/Acetone, 6:4).

*N-[3-(4-Trifluoromethylphenyl)-4-oxo-1,3-thiazolidin-2-yldene)-4-[5-(4-methylphenyl)-3-(trifluoro-methyl)-1H-pyrazol-1-yl]benzenesulfonamide* (**2e**). Light red powder, yield 67%, mp: 234–235 °C). ^1^H-NMR (400 MHz, CDCl_3_/TMS, δ ppm): 2.38 (3H, s, Ar-C**H**_3_); 4.08 (2H, s, S-C**H**_2_); 6.74–7.85 (13H, m, Ar-**H**). FT-IR ν _max_. (cm^−1^): 3471 (-OH, H_2_O); 1743 (C=O); 1327 and 1147 (SO_2_). Anal. for C_27_H_18_F_6_N_4_O_3_S_2_. 3/2 H_2_O: Found: C, 49.47; H, 3.25; N, 8.59%. Calc.: C, 49.77; H, 3.25; N, 8.59%. R*_f_*: 0.74 (PE/Acetone, 5:5).

### 3.3. Biological Activities

Locally bred balb/c mice of both sexes (30–35 g) were purchased from the animal breeding laboratories of Inonu University (Malatya, Turkey). The animals were fed a standard pellet diet and water *ad libitum* in a temperature-controlled room. On the day before the treatments, food was withdrawn, but the animals were allowed free access of water. The allocation of animals to different groups was randomized, and the experiments were carried out under blind conditions. Mice used in the present study were cared for in accordance with the directory of the Inonu University Animal Care Unit, which applies the guidelines of the National Institutes of Health on laboratory animal welfare. Procedures involving animals and their care were conducted in conformity with international laws and policies and animal studies accepted by Inonu University Ethical Council (2011/A17). 

Test samples, suspended in a mixture of distilled water and 0.5% sodium carboxymethyl cellulose (CMC), were given orally to the animals. The control group animals received the same experimental handling as those of the test groups, except that the drug treatment was replaced with appropriate volumes of the dosing vehicle. Either celecoxib [25 mg/kg, body weight (bw)] or ASA (200 mg/kg, bw) in 0.5% CMC was used as reference drug. 

In the pharmacological studies, in order to avoid wasting mice for inactive compounds, we employed a two-step activity screening model. For the preliminary activity screening, each test group was composed of four mice. All test drugs were administered to mice at doses of 100 mg/kg bw. Test compounds that possessed more than a 15% inhibitory effect in any of the measurement ranges were selected for further evaluation of the activity–dose relationship in two different doses (50 and 200 mg/kg). Because of the daily changing circumstance, we used another control group for each experiment. 

#### 3.3.1. Anti-inflammatory Activity, Carrageenan-Induced Oedema

For the determination of the effects on carrageenan-induced paw oedema, the modified method of Kasahara *et al.* was employed [37]. One hour after the oral administration of either test sample or dosing vehicle, each mouse was injected with a freshly prepared (0.5 mg/25 μL) suspension of carrageenan (Sigma, St. Louis, MO, USA) in physiological saline (154 mM NaCl). The subplantar tissue of the right hind paw was the injection site for all mice. The contralateral paw (left hind paw) was injected with 25 μL saline solution as the internal control. Paw oedema was measured every 90 min for 6 h after induction of inflammation with a pair of dial thickness gauge callipers (Ozaki Co., Tokyo, Japan). The difference in footpad thickness between the right and left foot indicated the degree of inflammation for each mouse. The change in paw volume, either increase or decrease, was calculated and compared with the control group (dosing vehicle) and analyzed using statistical methods. 

Percent inhibitory effects were estimated according to the following equation, where *n* was the average difference in thickness between the left and right hind paw of the control group and *n*′ was that of test group of animals:

Inhibition (%) = [(n − n’)/n] × 100

Celecoxib was used as a reference compound and administered at 25 mg/kg, bw. 

#### 3.3.2. Analgesic Activity, Koster Test

One hour after oral administration of test sample, each mouse was injected intraperitoneally with 3% (w/v) acetic acid solution (0.1 mL/10 g, bw). Starting 5 min after the acetic acid injection, the number of muscular contractions on mice were counted for a period of 10 min. A significant reduction in the number of writhings by any treatment as compared to the number of writhings in control animals was considered a positive analgesic response. The analgesic activity was expressed as a percentage change from writhing controls. ASA was used as a reference compound and administered at 200 mg/kg [38].

#### 3.3.3. Gastric–Ulcerogenic Effect

Eight hours after the treatments of the mice with synthesized compounds at a dose of 100 mg/kg bw for analgesic activity tests they were killed under deep ether anaesthesia and their stomachs were removed. Then the stomach of each mouse was opened through great curvature and examined for lesions or bleedings under dissecting microscope.

#### 3.3.4. Acute Toxicity

Animals employed in the carrageenan-induced paw oedema experiment were observed for 72 h, and the mortality rate was recorded for each group at the end of the observation period. 

#### 3.3.5. Antioxidant Activity, Lipid Peroxidation in Stomach and in Various Tissues (Liver, Kidney, Colon, Brain)

Only the animals which were administered 100 mg/kg of the test samples were subjected to this experimental process. Eight hours after the analgesic activity experiment, mice were killed under deep ether anesthesia, and their stomachs were removed. The stomach of each mouse was opened through great curvature and was examined for antioxidant activity. Lipid peroxidation was assessed by using the method of Ohkawa *et al.* [39] as modified by Jammal and Smith [40] and was expressed nmol of thiobarbituric acid (TBA)-reactive substances (TBARS)/g wet weight of tissue. ASA (200 mg/kg) and celecoxib (25 mg/kg) were used as standard drugs. However, LPO, GSH levels, MPO and SOD activities as tissue damage/antioxidant activity markers were measured in various tissues (liver, kidney, colon, brain). The animals who were administered 100 mg/kg and 200 mg/kg of the compound **1a** and celecoxib (25 mg/kg) as oral dose were subjected to this experimental process. After agents administered 72 hours, mice were killed and their tissues were removed.

#### 3.3.5.1. LPO and GSH Assays

Tissue samples were homogenized with ice-cold 150 mM KCl for the determination of LPO and glutathione (GSH) levels. The LPO levels were assayed by monitoring TBARS formation as described above. GSH measurements were performed using a modification of the Ellman procedure [28].

#### 3.3.5.2. MPO Assay

MPO is an enzyme that is found predominantly in the azurophilic granules of polymorphonuclear leukocytes (PMN). Tissue MPO activity is frequently utilized to estimate tissue PMN accumulation in inflamed tissues and correlates significantly with the number of PMN determined histochemically in tissues. MPO activity was measured in tissues in a procedure similar to that documented by Hillegass *et al*. [26]. 

#### 3.3.5.3. SOD Assay

SOD is a major antioxidant enzyme found in cells, which protect cells from oxidative stress. SOD activity in the tissue samples was measured according to the previously described method [27].

#### 3.3.5.4. Statistical Analysis of Data

Data obtained from animal experiments were expressed as means ± standard error (SEM). Statistical differences between the treatment and the control group of animals were evaluated by two-tailed Student’s *t* test. 

#### 3.3.6. Cancer Cell Growth Inhibitory Assay

The methodology of the NCI60 *in vitro* cancer screen can be found on-line at http://dtp.nci.nih.gov/branches/btb/ivclsp.html [29] and can be reviewed on their web site. 

#### 3.3.7. NS5B Inhibition Assay

All synthesized compounds were evaluated for their effect on HCV NS5B RNA dependent RNA polymerase activity in primer dependent elongation assays as previously described [30,31,32]. Activity of NS5B in the absence of the inhibitor but containing an equivalent amount of DMSO (control reaction) was set at 100% and that in the presence of the inhibitor was quantified relative to this control. 

##### Molecular Modeling

Molecular docking computations were carried out on a Dell Precision 470n workstation with the RHEL 4.0 operating system using Glide 5.5 (Schrodinger LLC, New York, NY, USA). 3D Structures of target compounds **1c** and **1d** were constructed using the fragment dictionary of Maestro 9.0 (Schrodinger LLC) and geometry was optimized by Macromodel program v9.7 (Schrodinger LLC) using the OPLS-AA force field with the steepest descent followed by truncated Newton conjugate gradient protocol. The X-ray crystal structure of NS5B polymerase in complex with MK-3281 (PDB ID: 2XWY) [33], with PF-868554 (PDB ID: 3FRZ) [34] with indole (PDB ID: 3TYV) [35] and with HCV-796 (PDB ID: 3FQL) [36] representing TP-I, TP-II, PP-I and PP-II pockets, respectively, obtained from the RCSB Protein Data Bank (PDB), were used in this study. The protein was optimized for docking using the “Protein Preparation Wizard” and “Prime-Refinement Utility” of Maestro 9.0. The grids were generated using bound inhibitor with the default parameters (the grids for site PP-III were generated using the extended box around HCV-796 bound at PP-II site of NS5B). The detailed extra precision Glide docking parameters were from our previous studies [32].

## 4. Conclusions

The carrageenan-induced paw edema assay in the rat is a test system often used for the testing of NSAIDs and COX-2 inhibitors in acute inflammatory processes. It has been described that the effect of selective COX- 2 inhibitors on carrageenan pleurisy in the rat over a time course ranging from 0–48 h after injection of the irritant. Generally, after 6 h, the COX-2 inhibitors were without effect whereas dual inhibitors still showed efficacy. This investigation produced great results and showed that efficacy by COX-2 inhibitors drastically depends on the time course of the inflammatory process: onset of inflammation, peak inflammation and resolution. COX-2 inhibitors showed anti-inflammatory activity early on during the onset of the inflammatory response coincident with the expression of COX-2 protein [41].

Our results show that compounds **1a**, **1d**, **1e**, **2d** exhibited higher inhibition of oedema formation in the carrageenan-induced rat paw oedema model. It is well documented that oedema produced by carrageenan is a biphasic event. If the inhibition is more effective in the first phase of carrageenan-induced oedema, then the effect is histamine and serotonin mediated. By contrast, if the inhibition is more effective in the second phase, then the effect is prostaglandin mediated [42]. Based on this principle, we speculate that compounds **1a** and **1d** exhibited anti-inflammatory effect through mechanisms involving suppression of PG. On the other hand, the compounds **1e** and **2d** mediated their effect through down-regulation of serotonin or histamine. However, further studies are warranted to demonstrate this mechanism. The anti-inflammatory activity of celecoxib derivatives was in the range of 19.4 to 36.2% for 90 min. Further, compound **1a** exhibited potent analgesic activity in comparison to the standard drug ASA (acetylsalicylic acid). 

This study also reports the analgesic activity and ulcerogenic risk of the new celecoxib derivatives. Few derivatives have been evaluated for potent anti-inflammatory activity devoid of ulcerogenic risk. All tested compounds showed significant reduction in ulcerogenic activity (0/5), whereas the standard drug ASA showed high severity index (3/5). The compounds **1a** and **2d** which exhibited noteworthy anti-inflammatory and analgesic activities, were found relatively safer for ulcerogenic risk than the other compounds. The structure-activity relationships seen for the anti-inflammatory activity were not confirmed for the analgesic activity. The analgesic activity of the reduced derivatives **1e** and **2d** were not significant when compared to the activity obtained by ASA or celecoxib. 

Our studies further identified compound **1c** as a potential lead for development of anti-HCV NS5B inhibitors. Sulfonylthiourea derivatives were found to be more potent than sulfonyl-iminothiazolidinones, while celecoxib the parent compound was the least active of all compounds examined in this investigation. In terms of growth inhibition, **1a** exhibited negligible cell growth inhibition at 10 µM concentration against variety of cell lines.

In conclusion, among the synthesized compounds, *N*-(ethylcarbamothioyl)-4-[5-(4-methylphenyl)-3-(trifluoromethyl)-1*H*-pyrazol-1-yl]benzenesulfonamide (**1a**), having an ethyl group at the *N*-position of the sulfonylthiourea structure, showed significant analgesic and promising anti-inflammatory activity with relatively reduced lipid peroxidation, and did not cause tissue damage in liver, kidney, colon or brain compared to control and celecoxib. Thus **1a** merits further attention for developing promising anti-inflammatory, analgesic, non-toxic compounds.
